# Auricular acupuncture for migraine: A protocol for systematic review and meta-analysis

**DOI:** 10.1097/MD.0000000000023036

**Published:** 2020-10-30

**Authors:** Yuqin Chen, Bingyang Liu, Weina Gong, Guoqiang Liu

**Affiliations:** Department of Rehabilitation Medicine of Linyi Central Hospital Linyi, Shandong Province, P.R. China.

**Keywords:** auricular acupuncture, migraine, meta-analysis and systematic review, protocol

## Abstract

**Introduction:**

Migraines are caused by neurological and vascular dysfunction, with a side or both sides of the head pain recurrent attack, often accompanied by nausea, vomiting, light, and sound allergy as the characteristics, is the clinical common disease, frequently occurring disease. The incidence of migraine is 8.4% to 28% worldwide (highest in Germany), and the lifetime incidence is about 14.0%. About 18.2% for women and 6.5% for men, About 23 percent of families have at least one migraine sufferer.^[1]^ It can occur at any age, and more than half of patients have headaches that interfere with work or school, while nearly a third may miss work or school because of the headache.^[2]^ Therefore, how to relief headache immediately and reduce the impact on life and work, becomes the basic clinical appeal of many patients. Analgesics are the main treatment for migraine in western medicine, many patients, who worried about the side effects of drugs, often take them only when the pain is unbearable, which can only treat the symptoms rather than the root causes.^[3]^ Auricular acupuncture as a form of acupuncture therapy which is proved to be effective in RCTs and very suitable for patients, has been used in patients who suffer from migraine for a long time, therefore a systematic review is necessary to provide available evidence for further study.

**Methods and analysis:**

The following databases will be searched from their inception to September 2020: Electronic database includes PubMed, Embase, Cochrane Library, Web of Science, Nature, Science online, VIP medicine information, and CNKI (China National Knowledge Infrastructure). Primary outcomes: Score of migraine symptoms. Additional outcomes: The overall effective rate. Data will be extracted by two researchers independently, risk of bias of the meta-analysis will be evaluated based on the Cochrane Handbook for Systematic Reviews of Interventions. All data analysis will be conducted by data statistics software Review Manager V.5.3. and Stata V.12.0.

**Results:**

The results of this study will systematically evaluate the effectiveness and safety of auricular acupuncture intervention for people with migraine.

**Conclusion:**

The systematic review of this study will summarize the current published evidence of auricular acupuncture for the treatment of migraine, which can further guide the promotion and application of it.

**Ethics and dissemination:**

This study is a systematic review, the outcomes are based on the published evidence, so examination and agreement by the ethics committee are not required in this study. We intend to publish the study results in a journal or conference presentations.

**Open Science Fra network (OSF) registration number:**

October 2, 2020 osf.io/q6arf. (https://osf.io/q6arf/.)

## 1 Introduction

Migraines are caused by neurological and vascular dysfunction, with a side or both sides of the head pain recurrent attack, often accompanied by nausea, vomiting, light, and sound allergy as the characteristics, is the clinical common disease, frequently occurring disease. The incidence of migraine is 8.4% to 28% worldwide (highest in Germany), and the lifetime incidence is about 14.0%. About 18.2% for women and 6.5% for men, about 23% of families have at least one migraine sufferer.^[1]^ It can occur at any age, and more than half of patients have headaches that interfere with work or school, while nearly a third may miss work or school because of the headache.^[2]^ Therefore, how to relief headache immediately and reduce the impact on life and work, becomes the basic clinical appeal of many patients. Analgesics are the main treatment for migraine in western medicine, many patients, who worried about the side effects of drugs, often take them only when the pain is unbearable, which can only treat the symptoms rather than the root causes.^[3]^ Acupuncture has many methods to treat migraine, positive effects, no adverse reactions, good, clear immediate analgesic effect, At the same time, it can effectively alleviate its accompanying symptoms. The World Health Organization (WHO) has included migraine in the recommended spectrum of diseases treated with acupuncture. In recent years, there have been a large number of clinical reports on acupuncture treatment of migraine.^[4]^ Auricular acupuncture as a form of acupuncture therapy which is proved to be effective in RCTs and very suitable for patients, has been used in patients who suffer from migraine for a long time, therefore a systematic review is necessary to provide available evidence for further study.

In recent years, acupuncture has been widely used in clinical and experimental studies of migraine, and its effectiveness has been fully proved.^[5]^ As a form of acupuncture, the auricular acupuncture has been used to relieve symptoms in patients with migraine, but its effectiveness and safety have not yet reached a definitive conclusion.^[6]^

Therefore, this research intends to adopt the method of system valuation and meta-analysis of the auricular acupuncture for migraine to evaluate the efficacy and safety.

## 2 Methods

### 2.1 Study registration

The protocol of the systematic review has been registered.

Registration: Open Science Fra network (OSF) registration number: October 2, 2020 osf.io/q6arf. (https://osf.io/q6arf/.) This systematic review protocol will be conducted and reported strictly according to Preferred Reporting Items for Systematic Reviews and Meta-Analyses (PRISMA)^[7]^ statement guidelines, and the important protocol amendments will be documented in the full review.

### 2.2 Inclusion and exclusion criteria for study selection

#### 2.2.1 Inclusion criteria

Inclusion criteria are all randomized controlled trials (RCTs), which main treatment of migraine is auricular acupuncture. The language of the trials to be included only Chinese or English.

#### 2.2.2 Exclusion criteria. Following studies will be excluded

1. Repeated publications
2. Review of literature and cases
3. Animal studies
4. Incomplete literature
5. Non-RCTs

### 2.3 Types of participants

The types of subjects included patients diagnosed with migraine, regardless of their degree and possible complications. All patients were treated with auricular acupuncture.

### 2.4 Interventions and controls

Interventions included treatment with auricular acupuncture. The control group only received conventional western medicine treatment. The routine treatment of each RCT may not be identical, but the use of auricular acupuncture is the only difference between intervention and control.

### 2.5 Types of outcome measures

#### 2.5.1 Main outcomes

1. Score of depression symptoms.

#### 2.5.2 Additional outcomes

1. The overall effective rate.

### 2.6 Search methods

#### 2.6.1 Search resources

**Figure 1:**
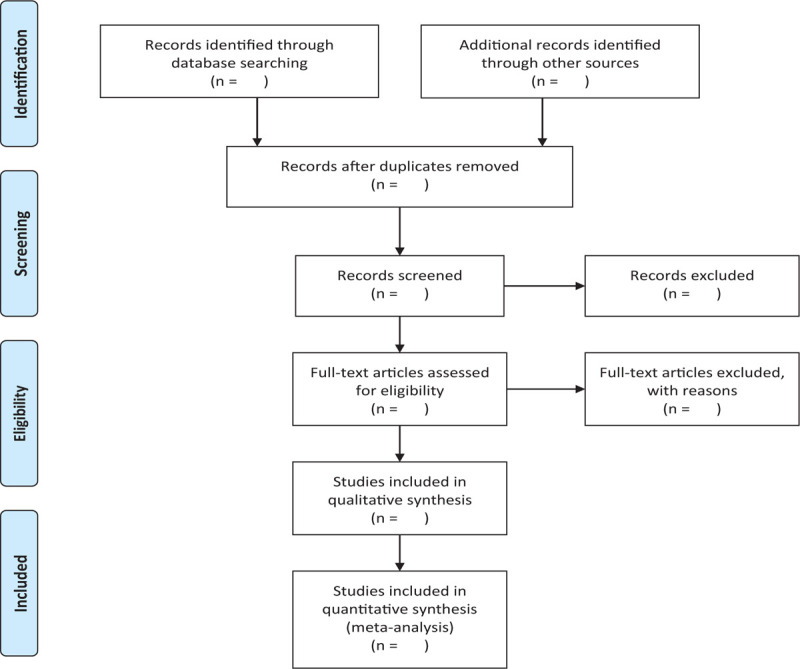
The research flowchart. This figure shows the Identification, Screening, Eligibility, and Included when we searching articles.

We will search the following electronic databases from their inception to September 2020: Electronic database includes PubMed, Embase, Cochrane Library, Chinese Biomedical Database, VIP medicine information, and CNKI (China National Knowledge Infrastructure). The research flowchart (Fig. 1).

#### 2.6.2 Search strategies

The following MeSH terms and their combinations will be searched:

1. auricular acupuncture;
2. RCT OR RCTs;
3. migraine. The research strategy (Table 1).

**Table 1:**
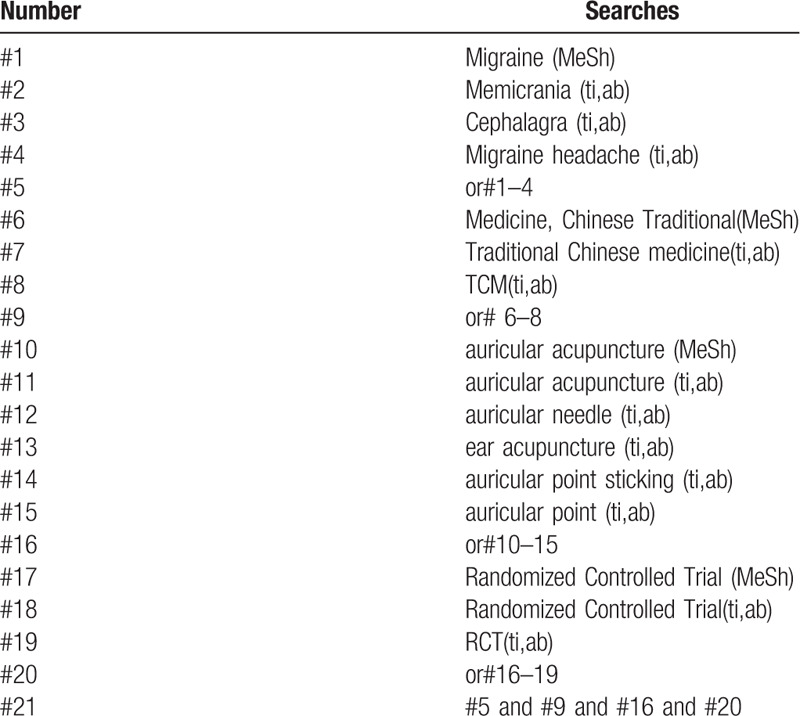
Search strategy sample of PubMed.

### 2.7 Data collection and analysis

#### 2.7.1 Studies selection

There will be two researchers (YC and BL) carry out the selection of research literature independently using Note-Express software. We will first make the preliminary selection by screening titles and abstracts. Secondly, we will download full text of the relevant studies for further selection according to the inclusion criteria. If there is any different opinion, two researchers will discuss and reach an agreement. If a consensus could not be reached, there will be a third researcher (GL) who makes the final decision. The details of selection process will be displayed in the PRISMA flow chart.

#### 2.7.2 Data extraction

Two researchers (YC and BL) will read all the included text in full, and independently extract the following information:

1. general information, including trial name, and registration information;
2. trial characteristic, including trial design, location, setting, and inclusion/exclusion criteria;
3. the characteristics of the participants, including age, race/ethnicity, course of illness, etc;
4. details of intervention, including acupoints, time of intervention, course of treatment, time of single treatment, etc;
5. details of comparison interventions;
6. out- comes as described under type of outcome measure section.

If we could not reach an agreement, a third researcher (GL) would make the final decision. One researcher (WG) would contact the corresponding author by telephone or e-mail for more information when the reported data were insufficient or ambiguous.

#### 2.7.3 Assessment of risk of bias

All the included studies will be evaluated based on the guidelines of Cochrane Handbook for Systematic Reviews of Interventions.^[8]^ The quality of each trial will categorized into “low,” “unclear,” or “high” risk of bias according to the following items: adequacy of generation of the allocation sequence, allocation concealment, blinding of participants and personal, blinding of outcome assessors, incomplete outcome data, selected reporting the results and other sources of bias (such as comparable baseline characteristic, inclusion, and exclusion criteria).

#### 2.7.4 Assessment of reporting biases

Reporting biases and small-study effects will be detected by funnel plot and Egger's test if there are 10 more studies included in this meta-analysis. For Egger's test, *P* value of <.10 was considered to indicate the exist of reporting biases and small study effects.

#### 2.7.5 Data analysis

We used Revman 5.3 software provided by the Cochrane collaboration to analyze the data. Binary outcomes will be summarized using risk ratio (RR) with 95% confidence interval (CI) for relative effect. Continuous outcomes will be summarized by using weighted mean difference (WMD) with 95% CI. We will use random-effect model (REM) for meta-analysis in this review according to research recommendations.^[9]^

Statistical heterogeneity will be assessed by X^2^ and *I*^2^ statistical tests. Where *P* value ≥.1 and *I*^2^ ≤ 50%, there is no obvious statistical heterogeneity among the studies. On the contrary, where *P* value <.1 or *I*^2^ > 50% indicates a considerable heterogeneity. Meta-analysis will be performed when the statistical heterogeneity is acceptable (*P* value ≥0.1 and *I*^2^ ≤50%), otherwise, subgroup analysis will be applied to explore the influence of potential factors on the outcome measures. We will conduct sensitivity analyses by omitting studies one by one in order to probe the impact of an individual study. If a meta-analysis cannot be performed, we will conduct descriptive analysis instead.

#### 2.7.6 Patient and public involvement

This is a meta-analysis study based on previously published data, so patient and public involvement will not be included in this study.

#### 2.7.7 Ethics and dissemination

Ethical approval will not be required as this is a protocol for systematic review and meta-analysis. The findings of this study will be disseminated to a peer-reviewed journal and presented at a relevant conference.

#### 2.7.8 Evidence assessed

The quality of evidence for this study will be assessed by “Grades of Recommendations Assessment, Development and Evaluation(GRADE) standard established by the World Health Organization and international organizations.^[10]^ To achieve transparency and simplification, the quality of evidence is divided into four levels in GRADE system: high, medium, low, and very low. We will employ GRADE profiler 3.2 for analysis.^[11]^

## 3 Discussion

According to epidemiological studies, the prevalence of migraine is currently about one in seven in the world.^[12]^ The prevalence was 9.3%, and more women than men.^[13]^ According to a community survey from Taiwan, about 14.4% of women and 4.5% of men suffer from migraines, 3.2% of the patients had chronic daily headaches.^[14]^ Earlier, severe migraines were identified by the World Health Report as the most disabling chronic disease, along with dementia, paralysis and severe mental illness.^[15]^ According to a 2013 data report, migraines rank sixth on the list based on the number of years of life lost to disability.^[16]^ The direct and indirect costs to patients of the disease, including healthcare costs, lost productivity and disability, can run into hundreds of millions of pounds each year. In addition, the migraine that breaks out repeatedly still can cause the pathological changes of cerebrum parenchyma.^[17]^ Therefore, active treatment and prevention of migraine has very important clinical significance. At present, the treatment of migraine is mainly reflected in the acute phase analgesia and the remission phase prevention. As the pathogenesis of migraine has not been clarified until now, the current situation of the diagnosis and treatment of migraine is still facing severe challenges. In particular, the problem of drug abuse in acute phase is more and more serious, which is dominated by non-steroidal anti-inflammatory drugs.^[18]^ Therefore, at the present stage, preventive treatment of migraine has been paid more and more attention by clinicians since 2010, emphasizing adequate preventive treatment so as to achieve optimal control of the disease.^[19]^ Now, on the basis of using drugs to control symptoms, more and more patients have to choose behavioral therapy and unconventional therapy to relieve symptoms, and the literature on the effectiveness of these therapies is extensive.

This systematic review will evaluate published RCTs evidence for the effectiveness and safety of auricular acupuncture for migraine. This study has several strengths, it may assist clinicians and patient treatment for migraine with guidelines. Clinical research will be conducted based on this systematic review protocol. In general, this review will be the first to evaluate the effects of auricular acupuncture on migraine outcomes. On this basis, a better treatment method can be established to provide a reliable basis for its wide application.

## Author contributions

**Conceptualization:** Yuqin Chen, Bingyang Liu,

**Data curation:** Yuqin Chen, Weina Gong

**Formal analysis:** Weina Gong,

**Methodology:** Yuqin Chen, Bingyang Liu,

**Project administration:** Yuqin Chen

**Resources:** Yuqin Chen, Bingyang Liu, Weina Gong

**Software:** Yuqin Chen, Bingyang Liu,

**Supervision:** Guoqiang Liu

**Writing – original draft:** Yuqin Chen

**Writing – review & editing:** Yuqin Chen
